# Relationship between Protein, MicroRNA Expression in Extracellular Vesicles and Rice Seed Vigor

**DOI:** 10.3390/ijms251910504

**Published:** 2024-09-29

**Authors:** Rouxian Wu, Bingxian Chen, Junting Jia, Jun Liu

**Affiliations:** Guangdong Key Laboratory for Crop Germplasm Resources Preservation and Utilization, Agro-Biological Gene Research Center, Guangdong Academy of Agricultural Sciences, Guangzhou 510640, China; chenbingxian@gdaas.cn (B.C.); jiajunting@agrogene.ac.cn (J.J.)

**Keywords:** rice, seed vigor, plant extracellular vesicles, miRNA

## Abstract

Plant extracellular vesicles are non-self-replicating particles released by living plant cells and delimited by a lipid bilayer. They contain a large amount of lipids, RNA, and proteins. Seed vigor plays an important role in agricultural production and preservation of germplasm resources. Extracellular vesicles with cross-species communication with bioactive molecules can resist pathogens, exhibit anti-aging properties, and perform other functions; however, its potential influence on seed vigor has not been reported. In this study, rice seeds with different germination percentages were used to extract extracellular vesicles, endogenous proteins, and RNA. Protein qualitative identification and miRNA differential analysis were performed to analyze the regulatory mechanism of extracellular vesicles on seed vigor. Results: The profiles of four miRNA families were found to be significantly different: osa-miR164, osa-miR168, osa-miR166, and osa-miR159. Protein correlation analysis predicted that extracellular vesicles might mediate the synthesis of the seed cell wall; glyoxic acid cycle and tricarboxylic acid cycle; non-specific lipid transfer; mitochondrial quality control; and other biological processes to regulate rice seed viability. In addition, cupin protein, phospholipase D, aldehyde dehydrogenase, seven heat shock proteins (especially BiP1 and BiP2), protein disulfide isomerase-like (PDI), thioredoxin, calnexin and calreticulin, glutathione transferase, and other proteins found in extracellular vesicles were closely related to seed vigor. This provides a novel direction for the study of the regulation mechanism of seed vigor.

## 1. Introduction

According to the International Society for Extracellular Vesicles’s (ISEV) latest research guidelines for extracellular vesicles (MISEV2023), the term “extracellular vesicles” (EVs) refers to particles that are released from cells, are delimited by a lipid bilayer, and cannot replicate on their own (i.e., do not contain a functional nucleus) [1]. At present, the study of extracellular vesicles mainly focuses on the study of cell supernatants and body fluid samples of mammals, including humans, mice, pigs, sheep, etc. The study of plant extracellular vesicles began in 1967 when researchers first observed, with electron microscopy, that carrot cells could secrete vesicles [2]. Twenty years earlier than in animals, extracellular vesicles were successfully isolated from the ectoplastid washing fluid of sunflower seeds fifty years after their first discovery [3]. Since these discoveries, plant extracellular vesicles have been isolated from many plant species, including ginseng, kudge, honeysuckle, licorice, turmeric, purpura, yam, ginger, garlic, broccoli, bitter melon, tomato, melon, grape, grapefruit, lemon, blueberry, citrus, coconut, cantaloupe, sunflower seed, oat, wheat, rice, and *Arabidopsis* [4]. Similar to mammals, plant extracellular vesicles are saucer-like or cup-shaped and are composed of large amounts of lipids, RNA, and proteins.

At present, the research on the biological function of plant extracellular vesicles mainly focuses on two aspects. First, for the plant itself, plant extracellular vesicles can resist the damage of pathogenic bacteria and play an immune role. They also play a role in intercellular communication and can transport proteins, lipids, and RNA molecules between plant cells. At the same time, they may maintain cell structure and function by secreting cell-wall-related proteins, removing harmful products from cells, participating in immune surveillance, and being involved in cell proliferation, differentiation, and response to stress and other stimuli [3,5]. Second, as a source of food or drugs for mammals and humans, existing studies have shown that plant extracellular vesicles can not only be used as therapeutic drugs, but also as carriers to transport therapeutic substances. They can also intervene and treat a variety of human diseases through cross-border transfer of RNA and other substances, with properties that include anti-inflammatory, anti-tumor, protection of regeneration (whiten effect), antiviral, anti-oxidative stress, anti-fibrosis, transcription regulation, liver protection, and other biological functions [3,4,6,7]. Recently, extracellular vesicles extracted from rice seeds have been found to contain hvu-MIR168-3p, which down-regulates the expression of mitochondrial electron transport chain complex I-related genes in human cells in vitro and in vivo and may help prevent GLUT1-related dysfunctions, including glucose metabolism, aging, and tumor immunology [8]. However, the effect of plant extracellular vesicles on rice seed viability has not been reported.

MicroRNA (miRNA) is a class of endogenous non-coding small RNA from eukaryotes. It is formed by splicing single-stranded RNA precursors with hairpin structures, and its length is about 20~24 nt. It plays a very important role in the regulation of plant gene expression [9,10]. Mature miRNAs will cooperate with AGO, Dicer, TRBP, PACT, and other elements to form miRISC, and they achieve silencing effects through two different mechanisms of action: degradation of mRNA and translation inhibition [11,12]. Plant miRNAs, which were first reported in 2002, are evolutionarily highly conserved, and their expression has obvious tissue specificity and time specificity during biological development, but they are similar among different plants [13]. Recent studies have shown that miRNA plays an important regulatory role in plant growth and development [14], biological and abiotic stress [15], and secondary metabolism [16], and has great potential in transboundary regulation. Some studies have suggested that miRNA is mainly transported through extracellular vesicles to achieve cross-border transport [17]. As a transboundary vector molecule, miRNA is involved in plant–pathogenic microorganism, plant–probiotic microorganism, and plant–animal regulation [18,19]. Plant seed development is a complex biological process (mainly including embryo, endosperm, seed coat development, and storage material accumulation), which is subject to multiple levels of genetic regulation. As an important genetic regulation mode, miRNA plays an important role in regulating the development, size, vigor, physical and chemical properties, and nutritional composition of seeds [13].

Seed vigor refers to the potential for rapid and orderly emergence of seeds and normal growth of seedlings under field conditions [20]. Seed vigor is of great significance in agricultural production and germplasm conservation. Seed vigor is a comprehensive trait [21], which depends not only on its own genetic factors but also on the environmental conditions during development and the storage conditions after seed maturity [22,23,24]. The process of seed quality decline and vitality reduction under natural conditions is called aging. Seeds after physiological maturity need to be stored in a dry, low-temperature environment. High temperatures and humid environments will strengthen seed respiration and metabolism and accelerate seed aging, deterioration, and mildew growth [25]. Taking rice seeds as test materials, some scholars concluded that adversity was not conducive to seed preservation, and the greater the damage of adversity conditions to seeds, the more obvious the decline of seed vitality. Low temperatures and suitable moisture contents could reduce the loss of vigor during seed storage [26,27].

Under natural conditions, the duration is longer, and the seeds of different rice varieties generally take 1 to 3 years to completely lose vitality. In this study, the differential expression of extracellular vesicles (bearing protein and miRNA) in rice seeds with a high germination percentage was compared with that of those that had been preserved for 3 years under natural conditions, and the mechanism of extracellular vesicles in regulating rice seed vitality was preliminarily investigated.

## 2. Results

### 2.1. Rice Seed Germination

**Figure 1:**
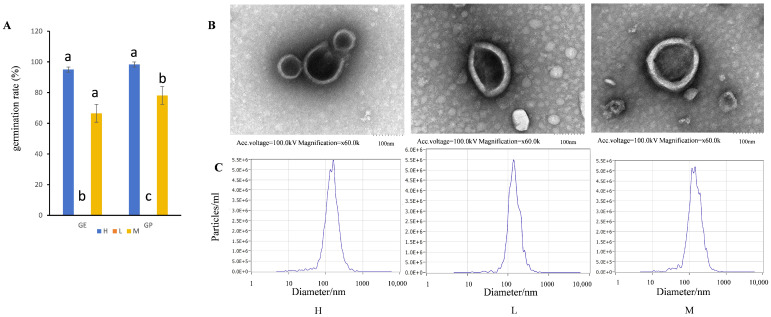
Extracellular vesicles were extracted from rice seeds with different vigor. (**A**) Germination rate of three batches of rice seeds (H samples were freshly harvested and threshed in November 2021, and they were sun-dried under natural light for one month; L samples were naturally dried for one month after harvest in November 2018 and stored in a net bag at room temperature, without any other treatment, exposed to natural air from December 2018 to December 2021; and M samples were newly harvested with their panicles in December 2021. The three batches of samples were processed in Baiyun District, Guangzhou). Different letters indicate significant level is *p <* 0.05. (**B**) 100 nm electron microscopy of EVs of three rice seeds. (**C**) Concentration of different particle sizes of EVs in three rice seeds.

Rice seed viability was assessed on the basis of germinability. According to International Rules for Seed Testing (1999), three batches of rice seeds in different states were tested for germination, and the seed germination rate was calculated on the 4th and 7th day (Figure 1A). The germination energy (GE) and germination percentage (GP) of the freshly harvested, threshed, and sun-dried seeds in 2021 (materials “H”) were 95% and 98.3%. The GP and GE of seeds harvested in 2018 and stored (materials “L”) were 0%. The GP and GE of the seeds newly harvested with their panicles in 2021 (materials “M”) were 66.5% and 78%. The seeds preserved under natural conditions for 3 years had no vitality, and the GP of newly harvested seeds with panicles were significantly lower than those of sun-dried seeds.

### 2.2. Extracellular Vesicles Extracted from Rice Seeds

EVs of rice seeds were isolated by an ultrafast centrifugation technique. Under transmission electron microscopy, EVs were irregularly circular, indented in the center, and had deeply stained, membrane-like structures on the periphery (Figure 1B), which were similar to the classic saucer-like and empty cup structures of exosomes. The size distribution of vesicles from the three samples was non-uniform; however, all vesicles had a diameter of less than 500 nm, suggesting a minimal presence of apoptotic bodies within these vesicles. Utilizing a nanoparticle tracking analyzer (NTA) for the assessment of exosome size distribution extracted from the cells revealed a range of 70.6~213.1 nm across the three samples, with peak sizes of 160.4 nm, 138.4 nm, and 143.3 nm (Figure 1C and Table 1). According to the evaluation criteria for EVs, this study has successfully isolated those from rice seeds with varying germination rates. Preliminary findings suggested the presence of EVs in both high-viability and non-viable seeds.

The correlation analysis results indicated that the correlation coefficients between the average particle size, peak particle size, and germination percentage all exceed 0.8, suggesting a significant association between particle size and germination percentage. The correlation coefficients between the initial concentration and the peak concentration of particle size and the germination percentage were all below 0.8, suggesting that the relationship between the concentration and the germination percentage is not statistically significant.

### 2.3. Proteins in EVs Derived from Rice Seeds

The EV proteins of H samples (three replicates) were identified by mass spectrometry. A total of 1231 peptides were identified, and the amino acid number of peptide segments was mainly between 7 and 20, accounting for 97%. The aforementioned peptides correspond to 406 effective proteins (Supplementary Table S1), and the relative molecular weight of the proteins ranges from 8.1 to 193.2 kDa with an average of 44.28 kDa.

**Figure 2:**
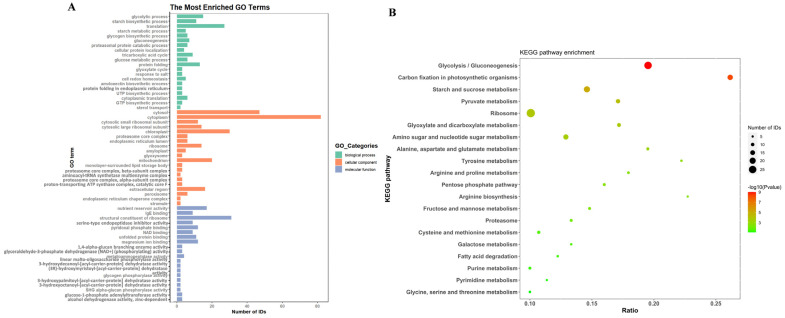
Functional annotation and enrichment analysis of proteins in EVs of H rice seeds. (**A**) Top 20 terms with the smallest *p* values in GO enrichment analysis. (**B**) Top 20 metabolic pathways in KEGG enrichment analysis.

The identified proteins were annotated by Gene Ontology (GO). Owing to the abundance of enrichment analysis terms, the top 20 with the smallest P values were selected for plotting analysis (Figure 2A). We observed that the primary biological processes (BP) implicated encompassed respiration (glycolytic process/tricarboxylic acid cycle/glyoxylate cycle); biosynthesis and metabolism of glucose and starch-like compounds; protein folding and localization; translation; cellular oxidative-reduction homeostasis; and sterol transport. The main cellular components (CC) involved include cytoplasm, ribosome, chloroplast, mitochondrion, endoplasmic reticulum lumen, proteasome core complex, amyloplast, glyoxysome, peroxisome, extracellular region, and others. In terms of molecular function (MF), it mainly plays the role of binding, dehydratase activity, dehydrogenase activity, phosphorylase activity, and regulator. The Kyoto Encyclopedia of Genes and Genomes (KEGG) was annotated for the identified proteins (Figure 2B). The top 20 metabolic pathways can be classified into 6 primary categories. Among these, two pathways were linked to cellular components (proteasome and ribosome), ten pathways were related to energy metabolism and carbohydrate metabolism (respiration), six pathways were associated with amino acid metabolism, one pathway was connected with carbon fixation in photosynthetic organisms, one pathway was involved in arginine biosynthesis, and one pathway was associated with fatty acid degradation. Among all the pathways, the ribosomal pathway, glycolytic/gluconeogenesis pathway, and starch/sucrose metabolism pathway exhibited the highest number of annotated proteins, indicating their crucial roles in the function of extracellular vesicles in rice seeds.

Forty-nine proteins with the highest Sequest HT score, most abundance, most enriched pathway or terms, and the highest association with seed viability were identified from extracellular vesicles of high-viability seeds (Table 2). We found that the expression levels and confidence of glutelin type-A/B, alpha-glucan-related enzymes, cupin, aspartic protease, and 63 k Da globulin-like protein were high in extracellular vesicles. After KEGG annotation, phosphoglucomutase (corresponding to two genes: *LOC_Os10g11140* and *LOC_Os03g50480*) was found to be involved in the most pathways (up to six). These were followed by aspartate aminotransferase (cytoplasmic), aldehyde dehydrogenase (NAD (+)), malate dehydrogenase, fructose-bisphosphate aldolase 1/3, glucose-6-phosphate isomerase (cytosolic A), pyrophosphate--fructose 6-phosphate 1-phosphotransferase subunit alpha/beta, and triosephosphate isomerase (cytosolic), which were involved in at least three pathways. Among them, the expressions of aspartate transaminase and triphosphate isomerase (cytosolic) were relatively high. The proteins associated with seed vigor include cupin protein, heat shock 70 k Da protein BiP1/BiP2, aldehyde dehydrogenase (NAD (+)), and protein disulfide isomerase-like 1-1/1-4/2-2. Among them, the reliability and expression of the cupin protein were high, and the other proteins were involved in at least two pathways except for the heat shock 70 k Da protein BiP1. In addition, non-specific lipid-transfer protein and glutathione S-transferase GSTF2 were the most abundant.

### 2.4. MicroRNA in EVs and Differential Expression Analysis

**Figure 3:**
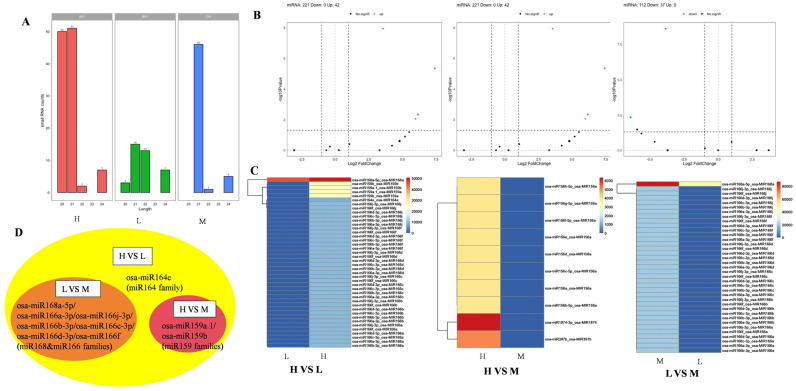
Analysis of miRNA and differential expression in EVs of three batches of rice seeds. (**A**) Expressed and annotated miRNA length distribution statistics. (**B**) miRNA expresses volcanic maps. The horizontal black dashed line indicates *p* = 0.05. The vertical dashed black lines indicate log2FC = −1 and log2FC = 1. (**C**) Differential miRNA expression heat map. Y-axis names are Mature miRNA names and Precursor miRNA names. (**D**) Relationship diagram of differentially expressed miRNAs in three rice seed EVs. The orange regions represent miRNAs with significant differences between materials L and M, the red regions represent miRNAs with significant differences between materials H and M, three color regions (orange, yellow, red) represent miRNAs with significant differences between materials H and L.

RNAs were isolated from EVs of rice seeds, and a total of 237 expressed MicroRNAs (miRNAs) were identified through small RNA-seq, data quality control, and comparative statistical analysis. A total of 187, 52, and 69 miRNA were identified from the three materials. There are 90 new predicted miRNA sequences, and the remaining annotated length distribution statistics were as follows (Figure 3A): the length of miRNA was 20–24 nt, and the length of miRNA was mostly 21 nt without 23 nt. In addition, osa-miR168a-5p and osa-miR5493 were the most read.

We found the following results by comparing the differential expression of miRNA in the EVs of rice seeds (Figure 3B): In dead seeds (material L) and high viability seeds (material H), 221 genes were screened, and 42 genes reached the drastically significant level (*p* < 0.01), all of which were up-regulated. A total of 203 differentially expressed miRNA genes were detected in high viability seeds (material H) and medium viability seeds (material M), and four of them reached a significant level (*p* < 0.05). A total of 112 differentially expressed miRNA genes were screened in medium active seeds (material M) and dead seeds (material L), and 37 miRNA genes reached a very significant level (*p* < 0.01), all of which were down-regulated. There were ten mature miRNAs with significant differential expression among the three materials, as follows: osa-miR159a.1, osa-miR159b, osa-miR164e, osa-miR166a-3p, osa-miR166b-3p, osa-miR166c-3p, osa-miR166d-3p, osa-miR166f, osa-miR166j-3p, and osa-miR168a-5p, which belong to the miR159, miR164, miR166, and miR168 families (Figure 3C). The above ten miRNAs were significantly differentially expressed (*p* < 0.01) in dead seeds (material L) and highly active seeds (material H) (Figure 3D). Osa-miR159a.1 and osa-miR159b were significantly differentially expressed (*p* < 0.05) in high-activity seeds (material H) and medium-activity seeds (material M), belonging to the miR159 family. Osa-miR166a-3p, osa-miR166b-3p, osa-miR166c-3p, osa-miR166d-3p, osa-miR166f, osa-miR166j-3p, and osa-miR168a-5p were significantly differentially expressed (*p* < 0.01) in medium-activity seeds (material M) and dead seeds (material L), which belong to the miR166 and miR168 families, respectively (Figure 3D). Among the two groups of highly significant expression (*p* < 0.01) alignments, osa-miR168a-5p had the highest expression level in live seeds, and only it had a relatively low expression level in dead seeds, and other differential miRNAs were not found in dead seeds (Supplementary Table S2).

The target genes of DE mature miRNA were searched online through the TarDB database. The target genes of 10 differentially expressed miRNAs were predicted, and a total of 90 gene transcripts were obtained. The predicted and experimentally verified target genes were separately analyzed for functional enrichment. In this study, we discovered (Table 3): (1) There were two predicted target genes of osa-miR164e. *Os03g0278000* was involved in the biological processes and molecular functions related to UDP-xylose metabolism, and *Os06g0590800* exerts related glycosyltransferase activity. (2) Osa-miR168a-5p had four target genes for predictive function. *Os02g0831600/Os04g0566500/Os02g0672200* were all involved in gene silencing (including post-transcriptional) by RNA. *Os07g0529000* was involved in the glyoxylic acid cycle, tricarboxylic acid cycle, and other related biological processes, the construction of glyoxylic acid cyclome and peroxisome, and the regulation of isocitrate lyase activity. (3) The miR166 family (osa-miR166a-3p/osa-miR166j-3p/osa-miR166b-3p/osa-miR166c-3p/osa-miR166d-3p/osa-miR166f) was predicted to have six functional target genes. *Os02g0676400/Os04g0571600* participated in xenobiotic transmembrane transport, xenobiotic detoxification by transmembrane export across the plasma membrane, and other related biological processes, and it exerted xenobiotic transmembrane transporter and antiporter activity. *Os03g0640800/Os10g0480200/Os03g0109400/Os12g0612700* have the molecular function of lipid binding. (4) The miR159 family (osa-miR159a.1/osa-miR159b) was predicted to have eight functional target genes. *Os01g0812000* was associated with the development of flowers (anthers and pollen). *Os02g0717400* was associated with mitochondrial fusion and localization (positive regulation of mitochondrial fusion/intracellular distribution of mitochondria/mitochondrion organization), mRNA binding, and other related GO-Terms (BP/CC/MF). *Os03g0578900/Os01g0812000/Os05g0490600* were implicated in the process of cell differentiation. 

*Os03g0683866* participated in the process of protein polyubiquitination as well as other associated biological processes and molecular functions (ubiquitin-protein transferase activity/ubiquitin protein ligase activity/ubiquitin conjugating enzyme activity/ubiquitin-dependent protein catabolic process). *Os05g0358700* was related to phospholipid catabolism and other processes (phospholipid catabolic process/phospholipase D activity).

## 3. Discussion

### 3.1. The Role of EVs in Seed Vigor

The availability of plant extracellular vesicles (EVs) is increasing, and it is becoming more common to isolate EVs from a variety of sources including fruits, vegetables, and Chinese medicinal materials [6,28]; however, there are limited reports on the extraction of EVs from rice or seeds. Recently, EVs have been isolated from rice seed bran (including the aleurone layer) samples using a centrifugation/filtration method, and their average particle size is approximately 97 nm [8]. In this investigation, EVs of whole rice seeds were extracted by ultrafast centrifugal method, similar to the approach employed by the previous study. The obtained size fell within the same subgroup (or grade), indicating the presence of EVs in rice seeds. In this study, EVs were also isolated from non-viable seeds, and there was no significant correlation between their concentration and bud rate. EVs are secreted by almost all living cells, including plant cells [6]. Therefore, the possibility that dead seeds contain living cells cannot be ruled out.

The impact of plant EVs on seed vigor remains unreported. In this study, we found that EVs extracted from seeds with different vigor had significantly different miRNA, and plant EVs were found to have functions such as resistance to pathogens, anti-aging, anti-inflammatory, and antioxidant stress, which were similar to the anti-aging mechanism of seeds. Microorganisms are one of the most important but often overlooked causes of seed aging and deterioration. Seed aging and deterioration are often related to microbial invasion. There are many kinds of microorganisms associated with seeds, including plant pathogens. Microorganisms that can participate in the aging and deterioration of seeds are ubiquitous, with various attack methods depending on the host. Water, temperature, and oxygen are the most important factors affecting microbial seed deterioration [29]. High-humidity environments are prone to causing pathogen invasion. For example, fungi can severely damage seeds during storage. They can grow within and on seeds at about 70% relative humidity, with increased growth rates at higher humidity levels [30]. Some research has indicated that Arabidopsis thaliana transports mRNA to gray mold via the release of EVs, which can impede gray mold infection following translation of the mRNA into protein [31]. Some scholars have analyzed EVs released into the environment by plant roots, showing that EVs can inhibit the spore growth of fungal pathogens [32]. Currently, it is considered that membrane lipid peroxidation and free radical accumulation in seeds are the two main reasons for seed vigor decline [33]. Previous studies have found that arabidopsis EVs transport lipids to participate in the immune response [34]. Some studies have pointed out that EVs secreted by young cells can improve the aging of elderly individual cells by reducing oxidative damage, revealing that EVs regulate GSH/GSSG through their own glutathione transferase (GST) activity and affect ROS accumulation in cells and organs, thus regulating the aging phenotype. Meanwhile, the ability of EVs to reduce lipid peroxidation levels has been demonstrated both in vitro and in vivo [35]. Ginger-derived EVs orally administered to normal mice were found to accumulate mainly in the liver and mesenteric lymph nodes, and they inhibit ROS production when acting on hepatocytes [36]. Ginger-derived vesicles have an inhibitory effect on Porphyromonas gingivalis [37]. Strawberry EVs could prevent oxidative stress in a dose-dependent manner by oral gavage in mice. The mechanism may be related to vitamin C and miRNA contained in strawberry EVs. Vitamin C acts as a natural free radical scavenger, protecting the body from oxidative stress [38]. In addition, recent studies have shown that the size of EVs is related to cardiac repair function and tumor invasion [39,40]. This is similar to the results of this study, in which particle size is related to bud rate. In conclusion, it is suggested that EVs in rice seeds have an effect on seed vigor, and the host seed viability may be related to the size of EVs.

### 3.2. Proteins Associated with Seed Vigor from EVs

In this study, a certain amount of cupin protein, phospholipase D, acetaldehyde dehydrogenase, seven heat shock proteins (especially BiP1 and BiP2), protein disulfide isomerase (PDI), thioredoxin, calnexin and calreticulin, and glutathione transferase were extracted from rice EVs. Studies have shown that these proteins directly or indirectly regulate seed vigor. Improved stability of oil in Arabidopsis seeds during storage and enhanced seed storage tolerance can be achieved by down-regulating the expression of the phospholipase D (*PLDα1*) gene [41]. The toxic effects of aldehydes produced by lipid peroxidation can be detoxicated by acetaldehyde dehydrogenase. Studies on the mutant of the rice acetaldehyde dehydrogenase gene (*osaldh7*) showed that *OsALDH7* plays an important role in seed maturation and longevity [42]. The deletion of *OsCDP3.10*, a candidate gene encoding cupin domain protein, which is used for the synthesis of storage protein 52 kDa globulin, significantly reduced the amino acid content in mature grains and early germinating seeds, thereby reducing the seed vigor of early germinating rice, including germination potential and seedling rate [43]. Seed storage proteins later become amino acids for embryonic and seedling development. During seed maturation, storage proteins are specifically synthesized in large amounts and deposited in the protein body through the endoplasmic reticulum (ER). The accumulation process is mediated by endoplasmic reticulum molecular chaperones such as BiP and PDI [44]. Previous studies have shown that heat shock proteins in the endoplasmic reticulum can help maintain normal protein folding function and play a role in stress resistance [45,46]. Heat shock proteins in the ER can enhance the anti-aging ability of seeds [47]. BiP, which belongs to the Hsp70 family, is a key chaperone involved in the folding of secreted proteins such as seed storage proteins in endoplasmic reticulum lumen. In rice, BiP1 is mainly expressed during seed maturation. Significant inhibition or overexpression of the BiP1 gene not only changes the seed phenotype and intracellular structure of endosperm cells, but also reduces the concentration of intracellular storage protein, starch accumulation, and grain weight [48]. In the process of seed endosperm formation, PDI, as a molecular chaperone, participates in the calponin/calreticulin cycle, one of the protein folding modes, and helps protein folding to form stable disulfide [49]. *PDIL1-1* controls endosperm development by regulating the amount and composition of proteins in rice seeds as a post-translational regulation, and its function loss can cause the accumulation of various seed proteins such as glucose/starch metabolism-related proteins and reactive oxygen species (ROS) to clear related unfolded proteins, thus affecting seed viability [50]. Glutathione transferase (GST) plays an important regulatory role in plant resistance to abiotic stress. It can reduce the damage of environmental pollution and oxidative free radicals in plants through mechanisms such as detoxification and antioxidant response, protect plant growth and development, and also participate in plant signal transduction and cytoderm synthesis [51]. Studies have found that, during the aging process of elm seeds, glutathione modification occurs at the cysteine site of glycolytic enzyme GAPDH1, which interacts with mitochondrial ion channel VDAC protein to cause the transformation of mitochondrial membrane permeability, leading to the release of pro-apoptotic factors, accelerating the occurrence of cell apoptosis, and regulating the vigor of elm seeds [52]. In this study, these proteins all come from EVs, and how EVs play a role in regulating seed vigor remains to be further explored, which also opens up a novel avenue for the study of seed viability.

### 3.3. Regulation of Seed Vigor by Four miRNA Families in EVs

Studies have shown that osa-miR164c and osa-miR168a are involved in the regulation of rice seed vigor [53]. Osa-miR164e and osa-miR168a-5p obtained in this study were extracted from EVs of rice seeds, and osa-miR164e was only expressed in active seeds (H vs. L was significantly different). Osa-miR168a-5p was highly expressed in viable seeds and was extremely low in dead seeds (H vs. L/L vs. M were significantly different). The miR164 family is unique to plants [54]. There are six members of the rice miR164 family. Osa-miR164c was proposed to influence seed vigor by regulating the expression of the target gene *PSK5*, and then successively affect the expression of the core gene *RPS27AA* and six types of functionally related genes [55]. Compared with the expression level of unaged rice seed embryos, osa-miR164e had a down-regulation function in aged rice seed embryos, indicating that both osa-miR164e and osa-miR164c were involved in the regulation of seed vigor [56]. Although osa-miR164c and osa-miR164e belong to the miR164 family, their sequences are uggagaagcaggguacgugca and uggagaagcagggcacgugag, respectively, with three different nucleotides. In this research, the target gene predicted for osa-miR164e was *UXS.* UDP-glucuronic acid decarboxylase (UXS), involved in xylan biosynthesis, is essential for normal cellulose deposition and the integrity of the cell wall. Meanwhile, proteins extracted from EVs of highly active seeds contained xylanase inhibitor protein 2 and XIP, xylose-isomerase, UDP-arabinopyranose mutase 1, and beta 1,3-glucanase, all of which were associated with the metabolism, biosynthesis, and modification of xylan, a component of the cell wall [57,58,59]. It has been shown that UDP-xylose is synthesized by UDP-glucuronide decarboxylase in the cytosol and Golgi lumen [60]. EVs originate from the Golgi apparatus or endoplasmic reticulum and are involved in cell wall synthesis [5]. Osa-miR164e is involved in the salt stress response of rice [61]. It is speculated that osa-miR164e and related enzymes act on the cell wall through EVs, thus indirectly regulating the high viability of rice seeds.

A considerable amount of miR168-3p was identified in EVs of rice seeds [8]. Sequence alignment revealed that miR168-3p was consistent with the osa-miR168a-5p sequence obtained in this study, and both were ucgcuuggugcagaucgggac. Thus, rice seed EVs contained large amounts of osa-miR168a-5p. miR168 is the only miRNA that has been shown to be a transboundary regulator of gene expression in plants and animals [62,63]. This is consistent with the conclusion that EVs can be transmitted across species [6]. For example, miR168 is mediated by extracellular vesicles between animals, plants, and pathogens. Some studies have shown that the overexpression of osa-miR168 (the sequence is consistent with that obtained in this study) leads to a higher germination rate of seeds than wild-type seeds [53]. Exogenous osa-miR168a-5p positively regulates seed vigor [64]. miR168 targets AGO1, a key component of the RNA-induced silencing complex (RISC). Therefore, changes in the regulatory signals of miR168-AGO1 lead to changes in the regulatory signals of multiple miRNA-target genes to regulate their expression and play a role in plant growth and environmental stress response [65]. Although the three target genes predicted by osa-miR168a-5p in this study all pointed to AGO1 protein, no AGO1 protein was found in the extracellular vesicles of high-viability seeds, and a small amount of AGO2 protein was identified. Recent studies have shown that AGO2 does have antiviral activity, mainly against tobacco rattle virus (TRV) and turnip mosaic virus (Tu MV) [66,67]. Whether the AGO2 protein plays a gene-silencing role in coordination with osa-miR168a-5p to affect seed vigor remains to be explored. Os07g0529000, another target gene predicted by osa-miR168a-5p in this study, is closely related to the tricarboxylic acid cycle and glyoxylic acid cycle. The extracted protein contains a considerable amount of isocitrate dehydrogenase (a key enzyme of the tricarboxylic acid cycle), isocitrate lyase (a key enzyme of the glyoxylic acid cycle), and energy-related proteins: ATP-dependent (S)-NAD(P)H-hydrate dehydratase, ATP synthase subunit alpha/beta, ADP-ribosylation factor 2, and other substances related to energy metabolism: pyruvate-related enzymes and sucrose synthases. These are associated with energy metabolism. In addition, studies have shown that the glyoxylic acid cycle plays an important role in plant response to stress [68]. Isocitrate dehydrogenase is involved in antioxidant stress [69]. This also indicates that relevant substances are involved in stress resistance. The expression levels of osa-miR168a-5p in living seeds were higher than those of other miRNAs. Therefore, it is speculated that in order to provide a large amount of energy to maintain seed vitality, osa-miR168a-5p may coordinate and initiate energy supply factors such as the glyoxylate cycle and tricarboxylic acid cycle for seed germination in the process of seed storage, so as to provide seeds with resistance to stress and maintain vitality.

There are 14 members of the osmiRNA166 family. In this study, there are six members with significant differences (H vs. L/L vs. M), and the sequences of these members are consistent: ucggaccaggcuucauucccc. The prediction function of the target gene is related to transmembrane transport and lipid binding. The related proteins extracted from EVs were non-specific lipid-transfer protein, calmodulin-2, and calnexin. Non-specific lipid transfer protein (nsLTP) is a kind of alkaline protein with low molecular weight. It can be secreted into the extracellular space to promote the movement of phospholipids between cell membranes. nsLTP is also involved in many key biological processes, such as pollen development, seed development, and cell wall extension, and plays an important role in biological and abiotic stress, plant resistance, and disease resistance [70,71]. nsLTP belongs to the polygenic family, and 52 members have been found in rice [72]. Currently, nsLTPs related to rice seed development (seed germination and germination rate) include *OsLTPL36* [73], *OsLTPL23* [74], and *OsLTPL18* [75], and *OsLTPL166* [70] suggested that nsLTPs played a key role in the regulation of seed vigor. nsLTP is a small peptide, each containing eight highly conserved cysteine residues in the form of C-XN-C-XN-CC-XN-CXC-XN-C-XN-XN-C. In addition, the N-terminal of nsLTPs has a hydrophobic signaling peptide, which is a short peptide chain that guides the transfer of newly synthesized nsLTPs to the secretion pathway. nsLTP has a calmodulin (CaM) binding site near the C-terminal, which regulates the interaction of nsLTPs with Ca^2+^ [71]. In this study, proteins extracted from EVs of living seeds contained nsLTPs and CaM. nsLTP consists of a three-dimensional structure of an internal hydrophobic cavity, and CaM has a spiral structure. OsmiRNA166 was significantly different between dead and live seeds, and its target class III homologous domain leu ZIP transcription factor (HD ZIP-III) had an α-helical hydrophobic surface [76]. Does structural similarity mean that OsmiRNA166, nsLTPs, and CaM are related in some way? It is speculated that osmiRNA166, nsLTPs, and CaM are related to each other and affect seed vigor through EVs.

There are 13 members of the osa-miRNA159 family, 5 of which were involved in this study. Only the first two members of osa-miR159a.1, osa-miR159b, osa-miR159c, osa-miR159d, and osa-miR159e were significantly differentially expressed in different active materials, and the sequence of these two members was the same: uuuggauugaagggagcucug. The expression of osa-miR159a.1 and osa-miR159b was significantly different in medium and high viability seeds, and it reached a very significant level in high viability and dead seeds. It can be seen that these two members play an important role in maintaining the high vitality of seeds. In this study, the positive regulation and distribution of osa-miR159a.1- and osa-miR159b-targeted mitochondrial fusion were investigated. Just as many studies have found that EVs carry mitochondrial protein [77], this study found that protein extracted from EVs contains 23 ribosomal proteins. Since the EVs were extracted from rice seeds and the seed coats were all white, it is likely that these ribosomal proteins were derived from mitochondria. Combined with the function of mitochondrial fusion, it is speculated that osa-miR159a.1 and osa-miR159b are related to the quality control of cellular mitochondria. Studies have shown that when lysosome function is impaired, cardiomyocytes can clear dysfunctional mitochondria and ubiquitinated proteins through EVs [78]. Research on quality control in plant mitochondria is still in its infancy, mainly focusing on autophagosomes [79]. The quality control method for whether plants have mitochondria-derived vesicles has not been reported. However, mitochondrial dysfunction related to ROS plays an important role in seed aging and deterioration. The large production of ROS induces the change and modification of mitochondria-specific proteins, which may participate in the process of mitochondrial deterioration and ultimately lead to the loss of seed vigor [80,81]. It is hypothesized that rice seeds employ EVs, osa-miR159a.1, osa-miR159b, and other signal transduction functions to act on damaged mitochondria in cells during storage, promote the fusion of damaged mitochondria, reduce the waste of energy and storage materials, and maintain high viability of rice seeds.

In summary, rice seed EVs transexpress osa-miR164e, osa-miR168a-5p, some family members of osa-miR166, osa-miR159a.1, osa-miR159b, and their corresponding proteins involved in mediating the synthesis of the seed cell wall, glyoxylic acid cycle and tricarboxylic acid cycle, non-specific lipid transfer, quality control of mitochondria, etc., so as to regulate the viability of rice seeds and maintain high seed vitality. In addition, the obtained differential miRNAs all have stress-resistant functions, and after the discovery of plant EVs, related studies mainly focus on improving immunity and resistance to stress. During the storage process, seeds are inevitably threatened by the external environment and pathogens, resulting in reduced vigor or even loss of vigor. When seeds are threatened, EVs are likely to act as signal transduction to activate relevant biological processes to reduce the harm caused by threats and ensure seed health. In addition, it is still necessary to investigate whether these miRNAs have a coordination role and whether they interact with other miRNAs or genes to regulate seed vigor. The mechanism and metabolic network of osa-miR164e, osa-miR168a-5p, some family members of osa-miR166, osa-miR159a.1, osa-miR159b, and their corresponding proteins involved in the regulation of seed viability were elucidated.

## 4. Materials and Methods

### 4.1. Seed Germination Test

The experiment started in 2021, and the three materials used were rice seed samples, among which H samples were freshly harvested and threshed in November 2021 and were dried under natural light for one month (the average temperature in December in the Baiyun District of Guangzhou was 20 °C, sunshine was 167 h/month, and humidity was 55%), L samples were naturally dried for one month after harvest in November 2018 and stored in a net bag at room temperature without any other treatment and exposed to natural air from December 2018 to December 2021. M samples were newly harvested with their panicles in December 2021. Each sample was repeated three times with a random selection of 100 grains each time. The germination test was carried out with filter paper (on paper) and culture dishes at room temperature (20–30 °C), and the 4-day germination energy (GE) and 7-day germination percentage (GP) were calculated. The germination standard was that the bud length was about half of the grain length, and the root length was equal to the grain length.

### 4.2. EVs Extraction from Rice Seeds

EVs of rice seeds were extracted by an ultrafast centrifugation method, and the EVs were detected by transmission electron microscopy and a nanoparticle tracking analyzer (NTA). Technical support is provided by PANOMIX in Jiangsu Province, China. A total of 6 g of rice seeds were removed from each portion, washed with sterile water, sterilized with 75% alcohol, and ground into powder. The enzyme solution for enzymatic hydrolysis was added at 50 °C for 6 h. The samples were centrifuged at 10,000 rpm for 1 h, the enzymic tissue slag was precipitated, and the supernatant was collected. The sample was moved to a new centrifuge tube and centrifuged at 2000× *g* at 4 °C for 30 min. The supernatant was carefully moved into a new centrifuge tube, and centrifuged at 10,000× *g*, 4 °C, 45 min to remove large vesicles. The supernatant was filtered with a 0.45 μm filter membrane, and the filtrate was collected. The filtrate was transferred to a new centrifuge tube, the overspeed rotor was selected, and it was centrifuged at 100,000× *g* at 4 °C for 70 min. After the supernatant was removed and re-suspended with 10 mL pre-cooled 1 × PBS, the overspeed rotor was selected and it was centrifuged again at 4 °C, 100,000× *g*, for 70 min. The supernatant was removed and re-suspended with 150 μL pre-cooled 1 × PBS. A total of 20 μL of extract was used for electron microscope detection, 10 μL extract was used for particle size analysis, and the remaining EVs were stored at −80 °C.

### 4.3. EVs Protein Extraction from Rice Seed

The proteins extracted from the EVs of the H sample were identified, the proteins were enzymatically decomposed into peptide segments, the peptide segments were identified by mass spectrometry, and then the possible proteins were derived. The proteins from the EVs of the A sample (three replicates were set up) were extracted by conventional protein extraction methods, and the obtained protein samples were sent to the relevant company’s enzyme extraction salt and mass spectrometry machine. The company uses the QExactive HF-X mass spectrometer with a full MS scan range of m/z 350–1500 for primary and secondary MS detection to generate raw MS data.The instrument to complete the mass spectrometry analysis was RIGOL L-3000 high performance liquid chromatography system purchased from Beijing RIGOL Technology Co., Ltd, Beijing, China. 

### 4.4. EVs RNA Extraction and miRNA Sequencing Analysis of Rice Seeds

A total of 700 μL of RNA lysate was added to the EVs and placed at room temperature for 5 min. A total of 140 μL of chloroform was added and swirled well for 15 s. It was incubated at room temperature for 3 min and centrifuged at 4 °C at 12,000× *g* for 15 min. The upper water phase was transferred to a new EP tube, 1.5 times the volume of anhydrous ethanol was added, and it was mixed well. A total of 700 µL of the mixture was absorbed and transferred to the RNeasy adsorption column which comes from RNAprep Pure Plant Total RNA Extraction Kit (DP432) provided by TIANGEN in Beijing, China, centrifuged at 8000× *g* at room temperature for 15 s, and the filtrate was abandoned. The procedure was repeated for the remaining mixture. A total of 700 μL of Buffer RWT was added, centrifuged 8000× *g* at room temperature at 500 μL for 15 s, and the filtrated was discarded. A total of 500 μL of Buffer RPE washing adsorption column was added, centrifuged 8000× *g* at room temperature for 15 s, and the filtrate was discarded. A total of 500 μL of Buffer RPE washing adsorption column was added and centrifuged at room temperature at 8000× *g* for 2 min. The filtrate and collection tube were discarded. The adsorption column was transferred to a new 2 mL centrifuge tube, centrifuged at 12,000× *g* for 1 min for drying, then the filtrate was discarded and the tube was collected. The adsorption column was transferred to a new 1.5 mL centrifuge tube, 30 μL RNase-free water was added to the middle of the adsorption membrane, centrifuged at 8000× *g* for 1 min, and RNA was eluted and transferred immediately to −80 °C freezer.

The PE150 sequencing scheme was adopted in the small RNA sequencing library, and fastqc was used to evaluate the quality of the sequencing library(PRJNA1165973, https://www.ncbi.nlm.nih.gov/ (accessed on 27 September 2024)). Fastp was used for N base excision, q20 filtration, and adaptor excision. The bowtie short sequence alignment tool was used to compare the removal of rRNA, tRNA, and other NcRNAs in the Rfam library. Quantitative statistics of small RNAs were performed using miRDeep2 (https://github.com/rajewsky-lab/mirdeep2 (accessed on 20 December 2022 )). DESeq2 (https://bioconductor.org/ (accessed on 20 December 2022)) was used for differential expression analysis.

### 4.5. Data Analysis

The *Oryza* sativa database(http://golgi.gs.dna.affrc.go.jp/SY-1102/rad/ (accessed on 17 March 2022)) was used for the proteins in this study. Proteome Discoverer2.4 software was used to search the database. Functional annotation and analysis of proteins were performed by Gene Ontology(http://geneontology.org/ (accessed on 18 October 2022)) and KEGG(https://www.genome.jp/kegg/ (accessed on 18 October 2022)). The reference genome of miRNA sequencing was the Nipponbare genome MSU7, mirbase-22-release (https://rice.uga.edu/ and http://www.mirbase.org/ (accessed on 22 December 2022 )). The target genes of DE mature miRNA were searched online through the TarDB database(http://www.biosequencing.cn/TarDB/ (accessed on 31 January 2023)). The predicted target genes were subjected to Gene Ontology(http://geneontology.org/ (accessed on 31 January 2023)) functional enrichment analysis. With *p*. adjust < 0.05 as the criterion, significant enrichment was screened, and enrichment terms were sorted according to *p*. adjust.

## 5. Conclusions

EVs were detected in both dead and live seeds, and the size of the EVs may be related to seed vigor. EV proteins mainly include the cupin protein, phospholipase D, acetaldehyde dehydrogenase, seven heat shock proteins (especially BiP1 and BiP2), protein disulfide isomerase like (PDI), thioredoxin, calcein and calreticulin, glutathione transferase, etc., which may be related to rice seed vigor. These proteins will open up new ideas for studying the regulation mechanism of seed vigor. There are several miRNA families related to vigor in the EVs of rice seeds: osa-miR164e, osa-miR168a-5p, osa-miR166a-3p, osa-miR166j-3p, osa-miR166b-3p, osa-miR166c-3p, osa-miR166d-3p, osa-miR166f, osa-miR159a.1, and osa-miR159b. The miRNA family members and their corresponding proteins are likely to regulate and maintain rice seed vigor through EVs mediating the synthesis of the seed cell wall, glyoxylic acid cycle and tricarboxylic acid cycle, non-specific lipid transfer, and mitochondrial quality control.

## Figures and Tables

**Table 1:** Results of NTA experiment on EVs of rice seeds with different vigor.

Rice Seed	Mean Diameter/nm	Concentration (Particles/mL)	Diameter/nm	Peak Concentration (Particles/mL)
H	157.7 ± 68.8	1.60 × 10^−11^	160.4	5.50 × 10^−6^
L	152.4 ± 56.3	5.00 × 10^−11^	138.4	5.50 × 10^−6^
M	156 ± 70.2	4.20 × 10^−11^	143.3	5.20 × 10^−6^

**Table 2:** Prominent proteins in EVs of highly viable seeds.

Accession	Description	Note
Q09151	Glutelin type-A 3	Highest Sequest HT score and most abundance
P14614	Glutelin type-B 4	Highest Sequest HT score and most abundance
P07728	Glutelin type-A 1	Highest Sequest HT score and most abundance
P07730	Glutelin type-A 2	Highest Sequest HT score and most abundance
A0A0P0W399	Alpha-1,4 glucan phosphorylase (Fragment)	Highest Sequest HT score and most abundance
Q653V7	Probable alpha-glucosidase	Highest Sequest HT score and most abundance
Q01401	1,4-alpha-glucan-branching enzyme, chloroplastic/amyloplastic	Highest Sequest HT score and most abundance
		Most enriched pathway or terms
Q852L2	Cupincin	Highest Sequest HT score and most abundance
		seed vigor correlation
Q75GX9	63 kDa globulin-like protein	Highest Sequest HT score and most abundance
Q42456	Aspartic proteinase oryzasin-1	Highest Sequest HT score and most abundance
P37833	Aspartate aminotransferase, cytoplasmic	Most enriched pathway or terms and
		most abundance
P48494	Triosephosphate isomerase, cytosolic	Most enriched pathway or terms and
		Most abundance
Q53MW2	Non-specific lipid-transfer protein	Most abundance
O82451	Probable glutathione S-transferase GSTF2	Most abundance
Q33AE4	Phosphoglucomutase (alpha-D-glucose-1,6-bisphosphate-dependent)	Most enriched pathway or terms
Q9AUQ4	Phosphoglucomutase (alpha-D-glucose-1,6-bisphosphate-dependent)	Most enriched pathway or terms
P17784	Fructose-bisphosphate aldolase 1, cytoplasmic	Most enriched pathway or terms
Q5N725	Fructose-bisphosphate aldolase 3, cytoplasmic	Most enriched pathway or terms
P42862	Glucose-6-phosphate isomerase, cytosolic A	Most enriched pathway or terms
Q2QXR8	Pyruvate kinase 2, cytosolic	Most enriched pathway or terms
Q0DCI1	Pyrophosphate-fructose 6-phosphate 1-phosphotransferase subunit alpha	Most enriched pathway or terms
Q0DD75	Pyrophosphate-fructose 6-phosphate 1-phosphotransferase subunit beta	Most enriched pathway or terms
Q2R1J8	30S ribosomal protein S4, chloroplastic	Most enriched pathway or terms
Q6K8B8	40S ribosomal protein S3a	Most enriched pathway or terms
Q75J18	60S ribosomal protein L13a-2, putative, expressed	Most enriched pathway or terms
Q2QNF3	60S ribosomal protein L2	Most enriched pathway or terms
Q6K4T1	60S ribosomal protein L27	Most enriched pathway or terms
Q6YY64	60S ribosomal protein L6	Most enriched pathway or terms
P0CH34	Ubiquitin-60S ribosomal protein L40-1	Most enriched pathway or terms
Q948T6	Lactoylglutathione lyase	Most enriched pathway or terms
Q7XUG1	Malate synthase	Most enriched pathway or terms
Q5JKW5	Malic enzyme	Most enriched pathway or terms
Q94JA2	Malate dehydrogenase	Most enriched pathway or terms
Q7XDC8	Malate dehydrogenase, cytoplasmic	Most enriched pathway or terms
Q6Z6M4	Isocitrate lyase	Most enriched pathway or terms
Q7F280	Isocitrate dehydrogenase [NADP]	Most enriched pathway or terms
Q0JBV4	Isocitrate dehydrogenase [NADP]	Most enriched pathway or terms
Q9LST8	Proteasome subunit beta	Most enriched pathway or terms
Q9LST4	Proteasome subunit beta	Most enriched pathway or terms
Q0DS36	Os03g0336100 protein	Highest Sequest HT score and most abundance
A0A0P0VUB4	Os03g0197300 protein (Fragment)	Highest Sequest HT score and most abundance
A0A0P0V679	Os01g0663400 protein (Fragment)	Highest Sequest HT score and most abundance
Q6ZK46	Os08g0127900 protein	Highest Sequest HT score and most abundance
Q6Z7B0	Heat shock 70 kDa protein BiP1	Seed vigor correlation
Q53RJ5	Heat shock 70 kDa protein BiP2	Seed vigor correlation
Q69P84	Aldehyde dehydrogenase (NAD(+))	Seed vigor correlation
A0A0P0UZH3	Phospholipase D (Fragment)	Seed vigor correlation
Q53LQ0	Protein disulfide isomerase-like 1-1	Seed vigor correlation
Q67IX6	Protein disulfide isomerase-like 1-4	Seed vigor correlation
Q942L2	Protein disulfide isomerase-like 2-2	Seed vigor correlation

**Table 3:** Functional annotation of ten differentially expressed miRNAs target genes.

Comparison Group	miRNA_ID	Rapdb	Description	Gene Count	Distinctiveness
H vs. L	osa-miR164e	*Os03g0278000/Os06g0590800*	D-xylose metabolic process	1	*p*. adjust < 0.05
			UDP-D-xylose biosynthetic process	1	*p*. adjust < 0.05
			UDP-glucuronate decarboxylase activity	1	*p*. adjust < 0.05
			UDP-glycosyltransferase activity	1	
			Intracellular membrane-bounded organelle	1	
			NAD+ binding	1	
			Lyase activity	1	
			Carboxy-lyase activity	1	
			Quercetin 7-O-glucosyltransferase activity	1	
			Quercetin 3-O-glucosyltransferase activity	1	
			Hexosyltransferase activity	1	
H vs. L, H vs. M	osa-miR159a.1	*Os11g0153400/*O*s03g0683866/Os02g0717400/Os03g0578900/Os12g0616400/Os05g0358700/Os01g0812000/Os05g0490600*	cell differentiation	3	*p*. adjust < 0.05
			Positive regulation of mitochondrial fusion	1	*p*. adjust < 0.05
			Intracellular distribution of mitochondria	1	*p*. adjust < 0.05
			Organelle localization	1	*p*. adjust < 0.05
			Organelle organization	1	*p*. adjust < 0.05
			Phospholipid catabolic process	1	*p*. adjust < 0.05
			Mitochondrion organization	1	
			Protein polyubiquitination	1	
			Regulation of catalytic activity	1	
			Multicellular organism development	1	
			Signal transduction	1	
			Ubiquitin-dependent protein catabolic process	1	
			Intracellular organelle	1	
			Phospholipase D activity	1	
			Ubiquitin conjugating enzyme activity	1	
			GTPase activator activity	1	
			Translation initiation factor activity	1	
			Ligase activity	1	
			Ubiquitin-protein transferase activity	1	
			Ubiquitin protein ligase activity	1	
H vs. L, H vs. M	osa-miR159b	*Os03g0578900/Os01g0812000/Os05g0490600*	Cell differentiation	3	*p*. adjust < 0.05
			Anther development	1	*p*. adjust < 0.05
			Pollen development	1	
			Flower development	1	
			mRNA binding	1	
H vs. L, L vs. M	osa-miR168a-5p	*Os02g0831600/Os04g0566500/Os02g0672200/Os07g0529000*	Gene silencing by RNA	3	*p*. adjust < 0.05
			Post-transcriptional gene silencing by RNA	2	*p*. adjust < 0.05
			Glyoxylate cycle	1	*p*. adjust < 0.05
			Glyoxysome	1	*p*. adjust < 0.05
			Carboxylic acid metabolic process	1	*p*. adjust < 0.05
			Tricarboxylic acid cycle	1	*p*. adjust < 0.05
			Isocitrate lyase activity	1	*p*. adjust < 0.05
			Regulation of secondary shoot formation	1	*p*. adjust < 0.05
			Peroxisome	1	
			Lyase activity	1	
H vs. L, L vs. M	osa-miR166a-3p/osa-miR166j-3p/osa-miR166b-3p/osa-miR166c-3p/osa-miR166d-3p/osa-miR166f	*Os04g0571600/Os02g0676400/Os03g0640800/Os10g0480200/Os03g0109400/Os12g0612700*	Lipid binding	4	*p*. adjust < 0.05
			Xenobiotic detoxification by transmembrane export across the plasma membrane	2	*p*. adjust < 0.05
			Xenobiotic transmembrane transport	2	*p*. adjust < 0.05
			Xenobiotic transmembrane transporter activity	2	*p*. adjust < 0.05
			Antiporter activity	2	*p*. adjust < 0.05

## Data Availability

All data supporting the findings of this study are available from the corresponding author on reasonable request.

## Supplementary Materials

The following supporting information can be downloaded at: https://www.mdpi.com/article/10.3390/ijms251910504/s1, Table S1: Comparison of standardized expression levels of different miRNAs in three materials, Table S2: 406 proteins were extracted and identified from extracellular vesicles of high vigor rice seeds.

## References

[1] Welsh J.A., Goberdhan D.C.I., O’DRiscoll L., Buzas E.I., Blenkiron C., Bussolati B., Cai H., Di Vizio D., Driedonks T.A.P., Erdbrügger U. (2024). Minimal information for studies of extracellular vesicles (MISEV2023): From basic to advanced approaches. J. Extracell. Vesicles.

[2] Wang L., Li J., Xu R., Chen L. (2020). Plant-derived exosomes: Research progress. J. Int. Pharm. Res..

[3] Zhao M., Li S.M., Zhang L., Cong M.H., Hu L.H., Qiao H.Z. (2021). Research progress of plant-derived vesicles and their biomedical applications. Acta Pharm. Sin..

[4] Cao M., Diao N., Cai X., Chen X., Xiao Y., Guo C., Chen D., Zhang X. (2023). Plant exosome nanovesicles (PENs): Green delivery platforms. Mater. Horizons.

[5] Zhang X., Lu Y., Zhang Y., Li X. (2023). Advances in Plant Extracellular Vesicles and Analysis Techniques. Biotechnol. Bull..

[6] Feng J., Xiu Q., Huang Y., Troyer Z., Li B., Zheng L. (2023). Plant-Derived Vesicle-Like Nanoparticles as Promising Biotherapeutic Tools: Present and Future. Adv. Mater..

[7] Li W., Xie R., Yang S., Ren L., Zhao R., Wang W. (2023). Research Progress of Plant Exosome-like Nanovesicles. Chin. J. Mod. Appl. Pharm..

[8] Akao Y., Kuranaga Y., Heishima K., Sugito N., Morikawa K., Ito Y., Soga T., Ito T. (2022). Plant hvu-MIR168-3p enhances expression of glucose transporter 1 (SLC2A1) in human cells by silencing genes related to mitochondrial electron transport chain complex I. J. Nutr. Biochem..

[9] Bartel D.P. (2004). MicroRNAs: Genomics, biogenesis, mechanism, and function. Cell.

[10] Voinnet O. (2009). Origin, Biogenesis, and Activity of Plant MicroRNAs. Cell.

[11] Tang G. (2005). siRNA and miRNA: An insight into RISCs. Trends Biochem. Sci..

[12] Liu W.-W., Meng J., Cui J., Luan Y.-S. (2017). Characterization and Function of MicroRNA*s in Plants. Front. Plant Sci..

[13] Liu Y., Yang J., Xu X., Xiong F. (2023). miRNA in Regulating Seed Development and the Response to Abiotic Stress in Plant: A Review. Chin. Agric. Sci. Bull..

[14] Jodder J. (2020). miRNA-mediated regulation of auxin signaling pathway during plant development and stress responses. J. Biosci..

[15] Vakilian K.A. (2020). Machine learning improves our knowledge about miRNA functions towards plant abiotic stresses. Sci. Rep..

[16] Vashisht I., Mishra P., Pal T., Chanumolu S., Singh T.R., Chauhan R.S. (2015). Mining NGS transcriptomes for miRNAs and dissecting their role in regulating growth, development, and secondary metabolites production in different organs of a medicinal herb, *Picrorhiza kurroa*. Planta.

[17] Middleton H., Yergeau É., Monard C., Combier J.-P., El Amrani A. (2020). Rhizospheric Plant–Microbe Interactions: miRNAs as a Key Mediator. Trends Plant Sci..

[18] Lu X., Zhang W., Zhang H., Lian Z., Chen H. (2022). Advances of miRNA-mediated regulatory roles in plant-microbe interaction. Chin. J. Biotech..

[19] Meng G., Liu B., Li Y., Ma Y., Wang X., Ma Y., Cai X. (2022). Advances in Cross-kingdom Regulation of Gene Expression Via miRNA in Animal and Plant. Anim. Husb. Feed. Sci..

[20] Sun Q., Wang J.-H., Sun B.-Q. (2007). Advances on Seed Vigor Physiological and Genetic Mechanisms. Agric. Sci. China.

[21] Gao H., Jing L., Chen L., Ju J., Wang Y., Zhu J., Yang L., Wang Y. (2016). Effects of elevated atmospheric CO_2_ and temperature on seed vigor of rice under open-air field conditions. Chin. J. Rice Sci..

[22] Fang Y., Song M. (2006). Research progress of seed vigor. Seed Sci. Technol..

[23] Liu Y., Wang T. (2012). Research progress of seed vigor. J. Maize Sci..

[24] Zhang H., Hu J. (2010). Seed Science.

[25] Mcdonald M.B. (1999). Seed deterioration: Physiology, repair and assessment. Seed Sci. Technol..

[26] Chen X., Zhang A., Han Z., Lu H., Ye Y., Zhou Q. (2014). Effects of Drying Temperature and Drying Time on Seed Moisture Content and Seed Vigor and Its Correlation Analysis at Different Havest Period in Rice. Southwest China J. Agric. Sci..

[27] Liao L., Xia S., Dong X., Lu H., Liu R. (2013). Effects of different storage conditions on seed germination ability of rice. China Seed Ind..

[28] Mu N., Li J., Zeng L., You J., Li R., Qin A., Liu X., Yan F., Zhou Z. (2023). Plant-Derived Exosome-Like Nanovesicles: Current Progress and Prospects. Int. J. Nanomed..

[29] Mcdonald M.B.J., Nelson C.J. (1986). Physiology of seed deterioration. Plant Growth Regul..

[30] Roberts E.H. (1972). Storage Environment and the Control of Viability.

[31] Wang S., He B., Wu H., Cai Q., Ramírez-Sánchez O., Abreu-Goodger C., Birch P.R., Jin H. (2024). Plant mRNAs move into a fungal pathogen via extracellular vesicles to reduce infection. Cell Host Microbe.

[32] De Palma M., Ambrosone A., Leone A., Del Gaudio P., Ruocco M., Turiák L., Bokka R., Fiume I., Tucci M., Pocsfalvi G. (2020). Plant Roots Release Small Extracellular Vesicles with Antifungal Activity. Plants.

[33] Ma S., Li J., Peng Z. (2011). Study on the Variation of MDA Content in the Seeds of *Pinus flexilis* James During Artificial Aging Course. Seed.

[34] Liu N.-J., Wang N., Bao J.-J., Zhu H.-X., Wang L.-J., Chen X.-Y. (2020). Lipidomic Analysis Reveals the Importance of GIPCs in Arabidopsis Leaf Extracellular Vesicles. Mol. Plant.

[35] Fafián-Labora J.A., Rodríguez-Navarro J.A., O’lOghlen A. (2020). Small Extracellular Vesicles Have GST Activity and Ameliorate Senescence-Related Tissue Damage. Cell Metab..

[36] Zhuang X., Deng Z., Mu J., Zhang L., Yan J., Miller D., Feng W., McClain C.J., Zhang H. (2015). Ginger-derived nanoparticles protect against alcohol-induced liver damage. J. Extracell. Vesicles.

[37] Sundaram K., Miller D.P., Kumar A., Teng Y., Sayed M., Mu J., Lei C., Sriwastva M.K., Zhang L., Yan J. (2019). Plant-Derived Exosomal Nanoparticles Inhibit Pathogenicity of *Porphyromonas gingivalis*. iScience.

[38] Perut F., Roncuzzi L., Avnet S., Massa A., Zini N., Sabbadini S., Giampieri F., Mezzetti B., Baldini N. (2021). Strawberry-Derived Exosome-Like Nanoparticles Prevent Oxidative Stress in Human Mesenchymal Stromal Cells. Biomolecules.

[39] van de Wakker S.I., Bauzá-Martinez J., Arceo C.R., Manjikian H., Blok C.J.B.S., Roefs M.T., Willms E., Maas R.G.C., Pronker M.F., de Jong O.G. (2023). Size matters: Functional differences of small extracellular vesicle subpopulations in cardiac repair responses. J. Extracell. Vesicles.

[40] Caponnetto F., Manini I., Skrap M., Palmai-Pallag T., Di Loreto C., Beltrami A.P., Cesselli D., Ferrari E. (2016). Size-dependent cellular uptake of exosomes. Nanomedicine.

[41] Devaiah S.P., Pan X., Hong Y., Roth M., Welti R., Wang X. (2007). Enhancing seed quality and viability by suppressing phospholipase D in Arabidopsis. Plant J..

[42] Shin J.-H., Kim S.-R., An G. (2009). Rice Aldehyde Dehydrogenase7 Is Needed for Seed Maturation and Viability. Plant Physiol..

[43] Peng L., Sun S., Yang B., Zhao J., Li W., Huang Z., Li Z., He Y., Wang Z. (2022). Genome-wide association study reveals that the cupin domain protein OsCDP3.10 regulates seed vigour in rice. Plant Biotechnol. J..

[44] Yasuda H., Hirose S., Kawakatsu T., Wakasa Y., Takaiwa F. (2015). Overexpression of BiP has Inhibitory Effects on the Accumulation of Seed Storage Proteins in Endosperm Cells of Rice. Plant Cell Physiol..

[45] Lindquist S., Craig E. (1988). The heat-shock proteins. Annu. Rev. Genet..

[46] Xu C., Bailly-Maitre B., Reed J.C. (2005). Endoplasmic reticulum stress: Cell life and death decisions. J. Clin. Investig..

[47] Rajjou L., Debeaujon I. (2008). Seed longevity: Survival and maintenance of high germination ability of dry seeds. Comptes Rendus Biol..

[48] Wakasa Y., Yasuda H., Oono Y., Kawakatsu T., Hirose S., Takahashi H., Hayashi S., Yang L., Takaiwa F. (2011). Expression of ER quality control-related genes in response to changes in BiP1 levels in developing rice endosperm. Plant J..

[49] Lu D.-P., Christopher D.A. (2008). Endoplasmic reticulum stress activates the expression of a sub-group of protein disulfide isomerase genes and AtbZIP60 modulates the response in *Arabidopsis thaliana*. Mol. Genet. Genom..

[50] Kim Y.J., Yeu S.Y., Park B.S., Koh H.J., Song J.T., Seo H.S. (2012). Protein disulfi de isomerase-like protein 1-1 controls endosperm development through regulation of the amount and composition of seed proteins in rice. PLoS ONE..

[51] Zhang X., Tao L., Qiao S., Du B., Guo C. (2017). Roles of Glutathione s-transferase in plant tolerance to abiotic stresses. China Biotechnol..

[52] Li Y., Wang Y., He Y.-Q., Ye T.-T., Huang X., Wu H., Ma T.-X., Pritchard H.W., Wang X.-F., Xue H. (2024). Glutathionylation of a glycolytic enzyme promotes cell death and vigor loss during aging of elm seeds. Plant Physiol..

[53] Zhou Y., Zhou S., Wang L., Wu D., Cheng H., Du X., Mao D., Zhang C., Jiang X. (2019). miR164c and miR168a regulate seed vigor in rice. J. Integr. Plant Biol..

[54] Mou G., Ji C., Xu D., Zhou G. (2013). Advances in plant miR164 family. Chin. Bull. Life Sci..

[55] Huang K.R. (2021). Elucidation of the miR164c-Guided Gene/Protein Interaction Network Controlling Seed Anti-Aging Ability in Rice. Master’s Thesis.

[56] Jiang X.C. (2011). Study on Correlation of miRNA and Rice (*Oryza sativa* L.) Seed Vigor. Master’s Thesis.

[57] Ma M., Hou C., Wang Q., Zhang C., Liu J., Weng X. (2011). Current progress on endoxylanase inhibitors in cereals. China Biotechnol..

[58] Liu X., Xie X., Zhu D., Chen H. (2020). Interaction between xylanase and XIP-type xylanase inhibitor protein: A review. Microbiol. China.

[59] Saqib A., Scheller H.V., Fredslund F., Welner D.H. (2019). Molecular characteristics of plant UDP-arabinopyranose mutases. Glycobiology.

[60] Xu S., Zhang M., Ye J., Hu D., Zhang Y., Li Z., Liu J., Sun Y., Wang S., Yuan X. (2023). Brittle culm 25, which encodes an UDP-xylose synthase, affects cell wall properties in rice. Crop J..

[61] Macovei A., Tuteja N. (2012). microRNAs targeting DEAD-box helicases are involved in salinity stress response in rice (*Oryza sativa* L.). BMC Plant Biol..

[62] Zhang L., Hou D.X., Chen X., Li D.H., Zhu L.Y., Zhang Y.J., Li J., Bian Z., Liang X.Y., Cai X. (2012). Exogenous plant miR168a specifically targets mammalian LDLRAPI: Evidence of cross-kingdom regulation by microRNA. Cell Res..

[63] Chen Z., Cai J. (2020). Study on osa-miR168a-5p Targeted Regulation of Human ADD1 and E2F2 Gene Expression. Med. Inf. Jun.

[64] Sun Y. (2019). Effects and the Relevant Proteomics of Exogenous miR168a on Seed Vigor of Hybrid Rice. Master’s Thesis.

[65] Zhou J., Zhang R., Jia X., Tang X., Guo Y., Yang H., Zheng X., Qian Q., Qi Y., Zhang Y. (2022). CRISPR-Cas9 mediated *OsMIR168a* knockout reveals its pleiotropy in rice. Plant Biotechnol. J..

[66] Ma X., Nicole M.-C., Meteignier L.-V., Hong N., Wang G., Moffett P. (2015). Different roles for RNA silencing and RNA processing components in virus recovery and virus-induced gene silencing in plants. J. Exp. Bot..

[67] Garcia-Ruiz H., Carbonell A., Hoyer J.S., Fahlgren N., Gilbert K.B., Takeda A., Giampetruzzi A., Ruiz M.T.G., McGinn M.G., Lowery N. (2015). Roles and Programming of Arabidopsis ARGONAUTE Proteins during Turnip Mosaic Virus Infection. PLOS Pathog..

[68] Lu Z. (2021). Origin of the Isocitratr Lyase (ICL) Gene in Plants and Functional Characterization of the Rice OsICL Gene. Master’s Thesis.

[69] Hao Z., Yuan J., Liu Y. (2012). Role of isocitrate dehydrogenase on oxidative stress in plants. Biotechnol. Bull..

[70] Wang W., Wang J. (2022). CRISPR/Cas9-mediated editing of the rice nsLTPs family gene *OsLTPL166*. Mol. Plant Breed..

[71] Ma Y., Yu B. (2021). nsLTPs Genes Involved in Plant Response to Stress: Research Progress. Chin. Agric. Sci. Bull..

[72] Boutrot F., Chantret N., Gautier M.-F. (2008). Genome-wide analysis of the rice and arabidopsis non-specific lipid transfer protein (nsLtp) gene families and identification of wheat nsLtp genes by EST data mining. BMC Genom..

[73] Wang X., Zhou W., Lu Z., Ouyang Y., Chol S.O., Yao J. (2015). A lipid transfer protein, OsLTPL36, is essential for seed development and seed quality in rice. Plant Sci..

[74] Li Q., Zhai W., Wei J., Jia Y. (2023). Rice lipid transfer protein, OsLTPL23, controls seed germination by regulating starch-sugar conversion and ABA homeostasis. Front. Genet..

[75] Li Y., Guo L., Cui Y., Yan X., Ouyang J., Li S. (2023). Lipid transfer protein, OsLTPL18, is essential for grain weight and seed germination in rice. Gene.

[76] Zhou Y., He N., Wei Q., Wang W., Xue X., Zhang S. (2010). Research Progress in HD-Zip Protein. Hubei Agric. Sci..

[77] Todkar K., Chikhi L., Desjardins V., El-Mortada F., Pépin G., Germain M. (2021). Selective packaging of mitochondrial proteins into extracellular vesicles prevents the release of mitochondrial DAMPs. Nat. Commun..

[78] Liang W., Sagar S., Ravindran R., Najo R.H., Quiles J.M., Chi L., Diao R.Y., Woodall B.P., Leon L.J., Zumaya E. (2023). Mitochondria are secreted in extracellular vesicles when lysosomal function is impaired. Nat. Commun..

[79] Jiang Y. (2021). Research progress of mitophagy in plants. Hubei Agric. Sci..

[80] Parkhey S., Naithani S., Keshavkant S. (2012). ROS production and lipid catabolism in desiccating Shorea robusta seeds during aging. Plant Physiol. Biochem..

[81] Li Y., Wang Y., Xue H., Pritchard H.W., Wang X. (2017). Changes in the mitochondrial protein profile due to ROS eruption during ageing of elm (*Ulmus pumila* L.) seeds. Plant Physiol. Biochem..

