# Supplementary material for: Relationship between Protein, MicroRNA Expression in Extracellular Vesicles and Rice Seed Vigor
Source: Int J Mol Sci. 2024 Sep 29;25(19):10504. doi: 10.3390/ijms251910504 (PMC11476841; doi:10.3390/ijms251910504)
Supplement: Supplementary file 1 [file ijms-25-10504-s001.zip › Table S1.pdf]

Table S1 Comparison of standardized expression levels of different miRNAs in three materials

miRNA	H	L	M
osa-miR164e	7	0	5
osa-miR168a-5p	40	4	30
osa-miR166a-3p	8	0	7
osa-miR166j-3p	8	0	7
osa-miR166b-3p	8	0	7
osa-miR166c-3p	8	0	7
osa-miR166d-3p	8	0	7
osa-miR166f	8	0	7
osa-miR159b	19	0	5
osa-miR159a.1	19	0	5
